# Implementation Science for Eliminating HIV Among Adolescents in High-Burden African Countries: Findings and Lessons Learned from the Adolescent HIV Prevention and Treatment Implementation Science Alliance (AHISA)

**DOI:** 10.1007/s10461-023-04038-8

**Published:** 2023-03-25

**Authors:** Susan Vorkoper, Nadia A. Sam-Agudu, Linda-Gail Bekker, Rachel Sturke

**Affiliations:** 1 Fogarty International Center, National Institutes of Health, Bethesda, MD USA; 2 Pediatric & Adolescent HIV Unit and International Research Center of Excellence, Institute of Human Virology Nigeria, Abuja, Nigeria; 3 Institute of Human Virology, Department of Pediatrics, University of Maryland School of Medicine, Baltimore, MD USA; 4 Department of Paediatrics and Child Health, University of Cape Coast School of Medical Sciences, Cape Coast, Ghana; 5 Desmond Tutu HIV Centre, University of Cape Town, Cape Town, South Africa

**Keywords:** implementation science, HIV, Africa, adolescent, young adult

## Abstract

Eliminating adolescent HIV in high-burden African countries depends on the success of implementing evidence-based interventions to reduce transmission and improve treatment outcomes. The Adolescent HIV Prevention and Treatment Implementation Science Alliance (AHISA) takes a collaborative approach to addressing key challenges and identifying and developing new areas of investigation to advance the adolescent HIV agenda. This special supplement represents the collective learning of the Alliance related to implementation science in the context of the adolescent HIV continuum of care from multiple African countries. Specifically, this series describes the current academic landscape of adolescent HIV and implementation science, such as the methodological use and utility of implementation measures and frameworks; addresses timely topics such as the use of innovative technologies for study adaptations in the context of the global COVID-19 pandemic; and explores opportunities to enhance adolescent-responsive approaches to HIV prevention and treatment using implementation science.

In 2021, 160,000 children under five years old were newly infected with HIV [1]. These children will join the 1.7 million children living with HIV today who will transition through adolescence in the next few years [2]. Eliminating HIV as a public health threat among adolescents in high-burden African countries depends heavily on successful implementation of evidence-based, contextually attuned interventions to reduce vertical and non-vertical transmission and improve treatment outcomes. Across the HIV continuum of care, adolescents are less likely to be tested and linked to care and treatment, have higher loss to follow-up, and have worse adherence and viral suppression rates than their adult counterparts [3–8]. This is happening despite the availability of evidence for appropriate and effective adolescent HIV treatment and care. This leads to the unacceptably high rates of AIDS related morbidity and mortality amongst adolescents and contributes to onward vertical transmission. Implementation science (IS) holds promise for (1) addressing these challenges by investigating major and multifactorial bottlenecks that impede effective implementation, (2) testing novel approaches to improve adolescent-centric health programming, and (3) determining the effect of implementation strategies to maximize the impact of evidence-based interventions and successfully taking them to scale [9].

This *AIDS & Behavior* special supplement provides insight into evidence generated by the Fogarty International Center’s Adolescent HIV Prevention and Treatment Implementation Science Alliance (AHISA). AHISA has adopted a solutions-driven, collaborative approach for adolescent HIV implementation research to address key challenges and identify and develop new areas of investigation in advancing the adolescent HIV agenda [10]. AHISA is composed of 26 teams of NIH-funded researchers, program implementers and policymakers working in 11 high HIV-burden African countries [11]. It uses a shared learning approach to identify gaps and provide opportunities for the application of IS to facilitate better utilization of scientific evidence in adolescent HIV programming and policy. Overall, AHISA focuses on IS priorities for adolescent HIV research, and with that, cross-collaboration and innovative solutions.

In this supplement, AHISA members present IS research findings related to the adolescent HIV continuum of care from multiple African countries. More specifically, articles in this series: (1) present data on components of the adolescent HIV continuum of care, including access to and utilization of testing and treatment and optimized integrated health services; (2) address timely topics such as the use of innovative technologies for study adaptations in the context of the global COVID-19 pandemic; (3) describe the current academic landscape of adolescent HIV and IS, such as the methodological use and utility of implementation measures and frameworks; and (4) explore opportunities to enhance adolescent-responsive approaches to HIV prevention and treatment using IS.

## Thematic Areas

### Application of Implementation Science in Adolescent HIV Research

In their broad scoping review, Vorkoper et al. [12] examine the application of IS methods in 44 adolescent HIV IS studies conducted in 13 African countries between 2010 and 2021. They note that only four (9%) studies used an IS framework, that acceptability and feasibility were the most evaluated implementation outcomes, and that 55% of studies described and tested at least one implementation strategy. Beima-Sofie et al. [13] critically review strategies used and implementation determinants and outcomes measured for a variety of evidence-based interventions in 36 studies conducted exclusively by AHISA teams. Similar to the findings by Vorkoper et al., most of the AHISA related studies focused on acceptability, reach, and feasibility; however, almost half (53%) used an IS framework/theory. This AHISA-specific analysis provides insight on the extent, utility, and outcomes of IS methods application in current adolescent HIV research conducted in high-burden African countries. Furthermore, it reflects the impact of AHISA’s collaborative IS knowledge and capacity building approach on its research network.

Subramanian and colleagues [14] reviewed the literature on integrated service delivery for adolescents living with HIV and created an IS-informed conceptual framework for integrated delivery of adolescent HIV care. This framework included a socioecological perspective, community and health care system linkages, and key components of adolescent-focused care. They applied the framework to ten studies and programs across seven African and South American countries and concluded that it can catalyze design and optimize implementation of adolescent HIV care.

Atujuna et al. [15] provide a commentary on the science of dissemination vis-à-vis implementation and discuss a USAID strategy to structure and target communication of research findings. The authors recommend approaches for embedding dissemination science in adolescent HIV IS studies, including developing a dissemination plan early in the process, engaging the relevant technical expertise, the application of methodological rigor and theory, active inclusion of youth voices, and use of digital platforms to maximize the reach of the disseminated content.

### Timely Topics and Innovative Approaches

Several articles addressed timely topics, such as, innovations used in adapting research and practice in the context of the global COVID-19 pandemic. Lowenthal et al. [16] and Ahmed et al. [17] highlighted the impact of COVID-19 on adolescent HIV prevention and treatment research and services. They conducted a survey of AHISA projects in nine African countries [16]. Most teams reported interruptions in study recruitment and follow-up, the need for pandemic-related protocol modifications, and introduction of remote research activities. Key lessons learned included the demonstration of resilience and resourcefulness among adolescents and young adults, the viability of remote service delivery strategies, and the need for research projects and health systems to respond to evolving contextual needs. Ahmed et al. [17] also reported on COVID-19 impact on adolescent services under three themes: service interruptions, service adjustments, and perceived individual-level health impacts. Their overall findings highlighted the need for further IS research to evaluate the effects of pandemic-related HIV service adaptations in high-HIV prevalence African countries. These lessons may also be applied to other disruptions common in the region such as natural disaster and conflict related displacement.

Innovative technologies are also vital to adolescent research and programming and are featured in the supplement. Despite an increase in the use of mHealth interventions in health research in low- and middle-income countries (LMICs), there is a paucity of studies evaluating their implementation in the context of adolescent HIV. Goldstein et al. [18] conducted a systematic review evaluating adolescent-focused mHealth interventions targeting adolescents and young adults along the HIV continuum of care in LMICs. They identified 27 mHealth interventions across the HIV continuum, notably with no mHealth interventions addressing transition from pediatric to adult care despite this being a particularly vulnerable phase in the continuum. They concluded that mHealth interventions have potential to remedy disparities along the HIV continuum of care for adolescents and young adults in LMICs, but larger, well-powered randomized trials are needed to provide evidence and support for scale up. Their findings also suggest that mHealth may be used within adolescent IS research as an implementation strategy in combination with evidence-based interventions to measure intervention effectiveness, costing, fidelity, and sustainability.

### Enhance Adolescent-Responsive Approaches Including Addressing Adolescent Mental Health and Wellbeing

A number of articles explored opportunities for IS to enhance adolescent-responsive approaches. These included best practices in adolescent engagement, tailoring programs, and peer support [14, 19, 20] as well as mental health and intersectional stigma [20–22].

Tahlil et al. [19] and Nelson et al. [20] highlighted the importance of youth inclusion when developing youth-related services. Through a crowdsourcing open call, Tahlil, et al. [19] solicited examples of how adolescents and young adults have been engaged in HIV research. They identified several adolescent and young adult engagement themes, including co-creating and co-leading the research process, building capacity across the community, and digital engagement. Across nine male adolescent sexual minority case studies, Nelson et al. [20] found that nearly all studies identified peer-models and youth-centered approaches as a strength. Specifically, the authors highlight these approaches as opportunities to facilitate authenticity and self-determination that can facilitate positive interactions between youth and service providers. Along with the article by Subramanian et al. [14], these articles emphasize the need for functional youth engagement in research, program, and policy development as an avenue to ensure that these initiatives meet the needs of the people they seek to serve.

Mental health is a critical driver of adolescent HIV outcomes [23]. The analysis by Boshe et al. [21] on research priorities related to adolescent mental health found that the prevalence and drivers of mental health disorders in this population were under researched. The highest ranked research priority gap was in the integration of mental health into existing HIV programs. Addressing these gaps would directly support the findings by Embleton et al. [22] that identified commonalities in adolescents’ intersectional stigma experiences in different contexts; this included the impact of stigma on the uptake and implementation of HIV prevention and treatment services. These conclusions were consistent with that of Nelson et al. [20] who highlighted the impact of stigma on health service availability and access for adolescent sexual minority men. All three articles emphasize the role of IS in addressing the “know-do gap” in adolescent mental health and in building the capacity of healthcare providers to provide stigma-free, rights-based and tailored care.

## Conclusion

This *AIDS & Behavior* supplement lays the groundwork for the application of IS in adolescent HIV research in high-burden countries. It provides data and first-hand lessons from ongoing and recently-completed studies. The information and research agenda presented establishes evidence and resources for practical approaches to support global adolescent health initiatives, such as the PEPFAR DREAMS, the Global Fund’s Strategic Investment in Adolescent Girls and Young Women, the World Health Organization’s Global Accelerated Action for the Health of Adolescents, Africa REACH and UNAIDS. Data and lessons learned shared in these articles will aid in identifying and addressing impediments to reducing HIV incidence and related mortality among adolescents. It also catalyzes and underpins the need for rigorous adolescent HIV-related implementation research to ultimately ensure global elimination of HIV in adolescents and young people.
